# A systematic review of anti-suicidal effects of sedative-hypnotics and cognitive behavioral therapy for insomnia

**DOI:** 10.1017/S1092852925000318

**Published:** 2025-06-02

**Authors:** Kyle Valentino, Kayla Teopiz, Sabrina Wong, Gia Han Le, Sebastian Badulescu, Danica Johnson, Roger Ho, Taeho Greg Rhee, Bing Cao, Joshua Rosenblat, Rodrigo Mansur, Roger S. McIntyre

**Affiliations:** 1 Brain and Cognition Discovery Foundation, Toronto, ON, Canada; 2 Department of Pharmacology and Toxicology, University of Toronto, Toronto, ON, Canada; 3 Mood Disorder Psychopharmacology Unit, University Health Network, Toronto, ON, Canada; 4 Institute of Medical Science, University of Toronto, Toronto, ON, Canada; 5 Department of Psychological Medicine, Yong Loo Lin School of Medicine; 6 Institute for Health Innovation and Technology (iHealthtech), National University of Singapore, Singapore; 7 Division of Life Science (LIFS), Hong Kong University of Science and Technology (HKUST), Hong Kong, P. R. China; 8 Department of Psychiatry, Yale School of Medicine, New Haven, CT, USA; 9 Department of Public Health Sciences, University of Connecticut School of Medicine, Farmington, CT, USA; 10 Key Laboratory of Cognition and Personality, Faculty of Psychology, Ministry of Education, Southwest University, Chongqing, P. R. China; 11 Department of Psychiatry, University of Toronto, Toronto, ON, Canada

**Keywords:** Sedative-hypnotics, depression, suicidality, CBT-I, orexin receptor antagonists

## Abstract

Suicide accounts for over 700,000 deaths per year globally and remains a public health priority. Evidence suggests that sleep-related interventions may be effective in reducing depressive symptom severity and suicidal thoughts in patients diagnosed with depression and comorbid insomnia. This study aims to systematically review the efficacy of sedative-hypnotics and/or cognitive behavioral therapy for insomnia (CBT-I) on measures of suicidality.

In accordance with Preferred Reporting Items for Systematic Reviews and Meta-Analyses (PRISMA) guidelines, PubMed, Medline, Cochrane Library, Embase, Scopus, and Web of Science were searched from inception to July 30, 2024. Studies were included if they (1) were randomized controlled trials (RCTs) and (2) reported on suicide-related measures associated with sleep interventions as a primary outcome, secondary outcome, or a safety measure. We endeavored to define and operationalize suicidality as suicidal ideation (SI), suicide attempts (SA), and suicide completion (SC). In cases where study authors failed to separate these three dimensions, the term “suicidality” was applied.

Eighteen studies were identified meeting inclusion criteria, comprised of studies investigating benzodiazepines (*n* = 2), Z-drugs (*n*=4), orexin receptor antagonists (ORAs) (*n*=8), and CBT-I (*n*=4). Zolpidem reduces SI as well as insomnia (linear association = 0.12, *p*<0.05) as evidenced by improvement on both the Columbia-Suicide Severity Rating Scale (C-SSRS) and the Scale for Suicide Ideation (SSI). ORAs were not associated with either an increase or decrease in suicidality. CBT-I alleviates SI in patients with insomnia (*t* = −3.35, *p*<0.05).

Effectively treating insomnia is associated with reduced SI. Available evidence suggests that Food and Drug Administration (FDA)-approved sedative-hypnotics do not increase the risk of suicidality.

## Introduction

According to the World Health Organization (WHO), the global suicide mortality rate is over 700,000 per year,^1^ with estimated reported SA of approximately 1.6 million per year.^2^ In the United States (U.S.), suicide is among the top 3 leading causes of death in individuals aged 15–34^3^ and among the top 9 leading causes of death in individuals 35–64.^3^ The economic burden of suicide and depression in the U.S. is $326.2 billion,^4^ and suicide prevention is a key public health priority across multiple countries.^1^

Rapid-acting anti-suicidal agents are critical for patients diagnosed with severe major depressive disorder (MDD) and experiencing SI. For example, esketamine was approved in August 2020 for adults with MDD at risk for suicide.^5^ Replicated evidence indicates that insomnia is associated with suicide-related outcomes.^6^ Consequently, it could be hypothesized that interventions that alleviate insomnia may have beneficial effects on measures of suicide. The FDA has approved many mechanistically dissimilar sedative-hypnotics in the treatment of insomnia including select antidepressants (e.g., doxepin), benzodiazepines (e.g., temazepam), “Z-drugs” (e.g., zolpidem, zaleplon, zopiclone), and dual orexin receptor antagonists (DORA; e.g., lemborexant, daridorexant, suvorexant).^7^ Evidence also suggests that CBT-I is effective in reducing depressive symptoms in persons with insomnia.^8^^,^^9^^,^^10^

Moreover, extant literature does suggest that select sedative-hypnotics and/or CBT-I may be effective in treating SI, SA, and SC, with potential mechanisms including an improvement in problem solving^11^ and nocturnal wakefulness.^12^ For example, coprescription of zolpidem during initiation of an antidepressant was beneficial in suicidal outpatients, especially in patients with severe insomnia.^11^ Pharmacovigilance data suggests that suvorexant, lemborexant, and daridorexant are significantly associated with lower odds of completed suicides compared to trazodone.^13^ Likewise, CBT-I has been reported to reduce measures of SI.^12^^,^^14^^,^^15^

Herein, this systematic review aims to identify and evaluate the anti-suicidal effects of benzodiazepines, Z-drugs, ORAs, and other FDA-approved sleep agents. In addition, the effect of CBT-I on measures of suicide is also evaluated.

## Methods

### Data sources and search strategy

The 2020 PRISMA guidelines were applied in this study.^16^ A systematic search was performed using the following electronic databases: PubMed, Medline, Cochrane Library, PsycInfo, Embase, Scopus, and Web of Science from inception through the end of July 2024. Additional studies were identified manually using Google Scholar. Search strings can be found in the supplementary material. A registered protocol does not exist for this review.

### Study selection

Studies were eligible for inclusion if they (1) were RCTs or (2) reported on if CBT-I or one of the following pharmacological sleep interventions were associated with suicide-related measures as either a primary outcome, secondary outcome, or as a safety measure: benzodiazepine, alprazolam, brotizolam, midazolam, triazolam, estazolam, loprazolam, lorazepam, lormetazepam, temazepam, flunitrazepam, flurazepam, nitrazepam, quazepam, zaleplon, zolpidem, zopiclone, eszopiclone, daridorexant, suvorexant, lemborexant, doxepin, quetiapine, secobarbital, benadryl, diphenhydramine, unisom, or doxylamine. Included drugs were either FDA-approved sedative-hypnotics or an agent used off-label for the treatment of insomnia. Studies were excluded if they (1) were not written in English; (2) were not peer reviewed; (3) did not have full-text availability.

Study screening and selection were conducted by two reviewers (KV). Titles and abstracts were initially screened for relevance, and full-text articles were subsequently assessed for eligibility. A second author (KT) cross-validated the screening and inclusion of retrieved studies.

### Data extraction

Published summary data from selected articles were independently extracted by KV and KT using a piloted data extraction form. Discrepancies were resolved via discussion with all additional authors. Information to be extracted was identified a priori and included (1) publication year, (2) sample size, (3) sample characteristics, (4) assessment tools, and (5) outcomes related to suicide. We endeavored to define and operationalize suicidality as SI, SA, and SC, reporting the aspect(s) observed in each identified study; however, in instances where study authors failed to separate these three dimensions, the term “suicidality” was applied.

### Quality assessment

The risk of bias was assessed for all included studies (Table 1). Consistent with the Cochrane Handbook for Systematic Review of Interventions,^17^ bias was evaluated based on the following areas: bias arising from the randomization process, bias due to deviations from intended interventions, bias due to missing outcome data, bias in the measurement of the outcome, bias in the selection of the reported result. Protocols were denoted as either “low risk,” “some concerns,” or “high risk.”

**Table 1.** Summary of Study Quality and Bias Assessment in Randomized Trials

Study	Bias arising from the randomization process	Bias due to deviations from intended interventions	Bias due to missing outcome data	Bias in the measurement of the outcome	Bias in the selection of the reported result
Gilbert et al. (2020)	Some concerns	Low risk	Low risk	Some concerns	Some concerns
Smith et al. (2002)	Low risk	Low risk	Low risk	Low risk	Low risk
McCall et al. (2019)	Low risk	Low risk	Low risk	Low risk	Low risk
Fava et al. (2006)	Low risk	Low risk	Low risk	Low risk	Low risk
Rumble et al. (2020)	Low risk	Low risk	Low risk	Low risk	Low risk
Krystal et al. (2007)	Low risk	Low risk	low risk	Low risk	Low risk
Fietze et al., (2022)	Low risk	Low risk	Low risk	Low risk	Low risk
Herring et al. (2016)	Low risk	Low risk	Low risk	Low risk	Low risk
Herring et al. (2016)	Low risk	Low risk	Low risk	Low risk	Low risk
Yardley et al. (2021)	Low risk	Low risk	Low risk	Low risk	Low risk
Uchimura et al. (2024)	Low risk	Low risk	Low risk	Low risk	Low risk
Rosenberg et al. (2019)	Low risk	Low risk	Low risk	Low risk	Low risk
Karppa et al. (2020)	Low risk	Low risk	Low risk	Low risk	Low risk
Moline et al. (2021)	Low risk	Low risk	Low risk	Low risk	Low risk
Kalmbach et al. (2022)	Low risk	Low risk	Low risk	Some concerns	Low risk
Yan Chan et al. (2022)	Low risk	Low risk	Low risk	Low risk	Low risk
Batterham et al. (2017)	Low risk	Low risk	Low risk	Some concerns	Low risk
Jernelov et al. (2021)	Low risk	Low risk	Low risk	Some concerns	Low risk

## Results

### Search results

**Figure 1.**
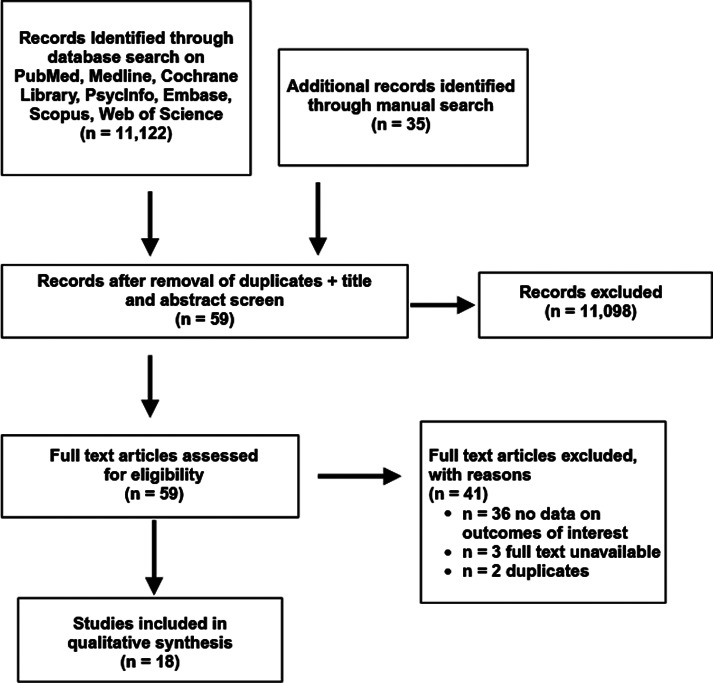
Study selection flow diagram.

The literature search yielded 11,122 studies. Following the removal of duplicates and screening of titles and abstracts, 59 articles were eligible for full-text screening against eligibility criteria. Following full-text screening, 36 studies were further excluded due to the absence of data related to the outcome(s) of interest. Study selection details are outlined in Figure 1. In total, 18 studies were included.

### Study characteristics

Sociodemographics, outcome measures, and results can be found in Table 2. Sample sizes ranged from 50 to 38,807 for the studies included. The ages of the participants ranged from 12 to 92. Patient diagnoses varied per study and included insomnia disorder, sleep–wake rhythm disorder (one study assessed this disorder comorbid with Alzheimer’s disease), post-traumatic stress disorder (PTSD), and MDD. According to reported numbers, females comprised 66.3% of the total population.

**Table 2.** Study Demographics and Outcomes

Study	Intervention	Diagnosis	Age range	Sample Size	% Female	Study length	Route of administration/dosage/treatment length	Suicide measure(s)	Outcome	Statistics
Gilbert et al. (2020)	-Alprazolam -Clonazepam -Lorazepam -Midazolam	PTSD	NR	38,807	NR	5 months	NR	Suicide-related behavior(s): ideation, attempt or death from suicide	Alprazolam and midazolam are associated with reduced risk of suicide-related behaviors relative to lorazepam and clonazepam	-HR (Suicide-Related Behaviors: alprazolam vs. clonazepam): 0.187 95% CI (0.039, 0.890) (*p*=0.0351) -HR (Suicide-Related Behaviors: alprazolam vs. lorazepam): 0.366 95% CI (0.142, 0.943) (*p* = 0.0373) -HR (Suicide-Related Behaviors: lorazepam vs. midazolam): 2.670 95% CI (1.430, 4.988) (*p* = 0.0021) -HR (Suicide-Related Behaviors: clonazepam vs. midazolam): 2.373 95% CI (1.089, 5.165) (*p* = 0.0297) -HR (Suicide-Related Behaviors: lorazepam vs. midazolam [FDR adjusted)]: 2.670 95% CI (1.430, 4.988) (*p* = 0.0315)
Smith et al. (2002)	-Clonazepam	Moderate depression	18–65	50	50%	18 weeks	-Oral -Fluoxetine: 20 mg, increased to 40 mg after 6 weeks if unresponsive per CGI-I and HAM-D criteria. Study drug: clonazepam 0.5 mg or placebo, dose was adjusted up to two tablets or down on the basis of tolerance and/or clinical improvement, during the first 2 weeks. The dose at 2 weeks continued for 12 weeks.	HAM-D	Clonazepam + fluoxetine cotherapy does not reduce suicide symptoms in MDD patients	-HAM-D core symptoms (fluoxetine monotherapy vs. fluoxetine + clonazepam combination therapy): 8.5±1.16 vs. 8.6±1.61 (*p*>0.05)
McCall et al. (2019)	-Zolpidem	-MDD -Insomnia -Suicide ideation	18–65	103	62%	8 weeks	-Oral -Nightly zolpidem-CR 12.5 mg or placebo for 8 weeks	C-SSRS	-When treatment for insomnia is successful, zolpidem is associated with a reduction in suicidal ideation in insomnia patients.	-No significant treatment effect was observed on SSI (least squares mean estimate=−0.56, SE=0.83, 95% CI=−2.19, 1.08), but the reduction in scores was significantly positively related to improvement in insomnia after accounting for the effect of other depression symptoms (*p*<0.002). -Cohen’s d (C-SSRS and zolpidem-CR (controlled release) SE=0.12, 95% CI=−0.50, −0.02, *p*=0.035) -The zolpidem-CR intervention was associated with a numerically greater reduction in the C-SSRS suicide ideation scores in participants with severe baseline insomnia (−0.41 ± 0.21), as compared with those with mild–moderate baseline insomnia (−0.08 ± 0.15).
Fava et al. (2006)	-Eszopiclone + Fluoxetine	-MDD -Insomnia	21–64	373	NR	8 weeks	-NR (route of administration) –10 weeks with fluoxetine hydrochloride (starting dose 20 mg; dose range: 20–40 mg/day) and randomized to also receive either eszopiclone 3 mg or placebo nightly for 8 weeks.	-HAM-D–17 -CGI-I -CGI-S	Eszopiclone + fluoxetine is not associated with increased suicide ideation in MDD patients.	NR
Rumble et al. (2020)	-Zolpidem	-MDD -Insomnia -Suicide ideation	18–65	103	62%	8 weeks	-Oral -Nightly zolpidem-CR 12.5 mg or placebo for 8 weeks	-SSI -C-SSRS	Zolpidem is associated with reduced insomnia severity, which was associated with a reduction in suicide ideation.	-Zolpidem-ER and insomnia severity: F(5,443) = 2.5, P = 0.03 -Suicide ideation and insomnia severity: F(1,448) = 9.78, P = 0.002
Krystal et al. (2007)	-Eszopiclone	-MDD -Insomnia	21–64	373	67.70%	8 weeks	-NR (route of administration) -Fluoxetine 20 mg each morning. Randomly assigned to adjunctive treatment with either eszopiclone cotherapy (ESZ+FLX group) or fluoxetine monotherapy (PBO+FLX group), administered nightly immediately before bed, for 8 weeks. At the end of 8 weeks, all patients continued open-label fluoxetine monotherapy treatment along with single-blind placebo (administered immediately before bed) for two weeks.	HAM-D–17	Eszopiclone is not associated with a reduction in suicide ideation.	-Change in baseline depression (placebo + fluoxetine vs. eszopiclone + fluoxetine): −0.37 (0.03) vs. −0.40 (0.03) (*p*= 0.4706)
Fietze et al. (2022)	-Daridorexant	Insomnia	18 – > 65	566	67.10%	3 months	-Oral daridorexant 50 mg, daridorexant 25 mg or placebo every evening for 12 weeks	Suicide attempt/ideation	Dariorexant is not associated with increased suicide-related outcomes in young adults with insomnia.	NR
Herring et al. (2016)	-Suvorexant	Insomnia	18 – > 65	2030	64.10%	3 months	-NR (route of administration) -Nightly administration -Patients were randomized to 3 months of treatment with suvorexant 40/30 mg, suvorexant 20/15 mg, or placebo. Doses differed by age to adjust for previously observed plasma exposure differences (<65 years: 40 mg or 20 mg; ≥65 years: 30 mg or 15 mg). Randomization was stratified by age category (nonelderly vs. elderly) and cohort. For the run-out at the end of treatment, half of the patients initially randomized to suvorexant were randomized to either continue on the same dose of suvorexant (suvorexant → suvorexant) or to switch to placebo (suvorexant → placebo), while patients initially randomized to placebo continued to receive placebo (placebo → placebo).	C-SSRS	Suvorexant is not associated with increased suicide ideation or attempts.	NR
Herring et al. (2016)	-Suvorexant	Insomnia	18 – > 65	1260	NR	3 months	-Oral -Daily -Patients were randomized to treatment with suvorexant 40/30 mg, suvorexant 20/15mg, or placebo. Doses differed by age to adjust for previously observed plasma exposure differences (<65: 40 mg or 20 mg; ≥65: 30 mg or 15 mg). Randomization was stratified by age category (non-elderly vs. elderly) in all trials and also by cohort.	C-SSRS	Suvorexant is not associated with increased suicide ideation or attempts.	NR
Yardley et al. (2021)	-Lemborexant	Insomnia	18–88	303	68.20%	12 months	-Oral -During treatment period 1 (first six months), subjects were randomized to either daily placebo, lemborexant 5 mg (LEM5). or lemborexant 10 mg (LEM10). During treatment period 2 (subsequent six months), all LEM5 and LEM10 subjects continued their originally assigned dose, whereas subjects in the placebo group in treatment period 1 were rerandomized to treatment with LEM5 or LEM10.	Suicide ideation/attempt	Lemborexent is not associated with increased suicide-related outcomes.	NR
Uchimura et al. (2024)	-Daridorexant	Insomnia	18 – > 65	490	49.60%	6 weeks	-Oral daridorexant 50 mg, daridorexant 25 mg, or placebo, every evening before bedtime, at least 2 h after the last meal, for 28 days	C-SSRS	Daridorexant is not associated with increased suicide ideation or attempts.	NR
Rosenberg et al. (2019)	-Lemborexant	Insomnia	> 55	1006	NR	2 months	-Oral –5 mg of lemborexant, 10 mg of lemborexant, 6.25 mg of zolpidem, or placebo -Participants were treated for 30 nights	C-SSRS	Lemborexant is not associated with increased suicide ideation or attempts.	NR
Karppa et al. 92020)	-Lemborexant	Insomnia	18 – > 65	949	68.20%	12 months	-Oral -Placebo or lemborexant (5 mg [LEM5] or 10 mg [LEM10]) nightly –12 months	C-SSRS	Lemborexant is not associated with increased suicide ideation or attempts.	NR
Moline et al. (2021)	-Lemborexant	-Alzheimer’s disease -Sleep wake rhythm disorder	60–90	60	57.00%	4 weeks	-NR (route of administration) -Subjects were randomized to placebo or one of four lemborexant treatment arms (2.5 mg, 5 mg, 10 mg, or 15 mg) once nightly at bedtime for 4 weeks	C-SSRS	Lemborexant is not associated with increased suicide-related outcomes as measured by the CSSRS.	NR
Kalmbach et al. (2022)	-CBT-I	Insomnia	18–92	126	78.90%	1 year	N/A	QIDS-SR16	Digital CBT-I reduces insomnia symptoms, which promotes suicide ideation alleviation and prevention.	PRODCLIN estimate of the indirect effect supported a significant indirect effect wherein CBT-I increased the likelihood of insomnia remission, which was associated with suicide ideation prevention (αβ = −3.20, 95% CI = −5.74 to −0.87)
Yan Chan et al. (2022)	-CBT-I	Insomnia	12–24	135	67.40%	6 months	N/A	The Depressive Symptom Inventory–Suicidality Subscale	Group CBT-I is associated with reduced suicide ideation.	Cohen’s d (suicide ideation: group CBT vs. email self-help): d = −0.64 (*p* = 0.01)
Batterham et al. (2017)	-CBT-I	-Insomnia -Subclinical depression symptoms	18–64	1149	74.00%	6 weeks	N/A	PSF	Online internet-based CBT-I is associated with a temporary reduction in suicide ideation post-intervention, but not at 6 months.	-Cohen’s d (PSF suicide-related outcomes post Test: SHUTi vs. HealthWatch): 0.13 *p*=0.007 -Cohen’s d (PSF suicide-related outcomes 6 months: SHUTi vs. HealthWatch): 0.08 (*p*=0.303) -PSF suicide-related outcomes (SHUTi vs. HealthWatch): F4, 459.5=2.2, (*p*=0.069)
Jernelov et al. (2021)	-CBT-I	Insomnia	> 16	522	66.00%	9 weeks	N/A	MADRS-suicidality	CBT-I is associated with reduced suicide ideation.	-Cohen’s d (suicide ideation) (CBT-I vs. placebo): 0.16, 95% CI (0.04, 0.29) -Linear mixed models analysis (suicide ideation and CBT-I): *t* = −3.35, df = 529.2, *p* = 0.001

Abbreviations: BSSI, Beck Scale for Suicidal Ideations, CBT-I, Cognitive Behavioral Therapy for Insomnia, CGI-I, Clinical Global Impression Improvement, CGI-S, Clinical Global Impression Improvement Severity Items, CI, confidence interval; C-SSRS, Columbia Suicide Severity Rating Scale, df, degrees of freedom, FDR, false discovery rate, HAM-D, Hamilton Rating Scale for Depression, HR, hazard ratio, LEM, Lemborexant, MADRS, Montgomery–Åsberg Depression Rating Scale, NR, not reported, PRODCLIN, PRODuct Confidence Limits for INdirect effects, PTSD, post traumatic stress disorder, PSF, psychiatric symptom frequency, QIDS-SR16, Quick Inventory of Depressive Symptomatology, SSI, Scale for Suicide Ideation, SHUTi, Sleep Healthy Using the Internet, zolpidem-CR, zolpidem controlled-release, zolpidem-ER, zolpidem extended-release.

### Benzodiazepines

Two RCTs studied the anti-suicidal effects of benzodiazepine medications.

Findings suggest that alprazolam and midazolam are associated with reduced risk of suicide-related behaviors (SRBs), defined as SI, SA, or SC. In PTSD patients, alprazolam was associated with fewer SRBs compared to clonazepam (Hazard Ratio (HR) 0.187 (95% CI [0.039, 0.890] *p* = 0.0351) and lorazepam (HR 0.366 (95% CI [0.142, 0.943] *p*=0.0373) over an average 6 month follow-up period.^18^ Likewise, it was observed that patients prescribed midazolam experienced fewer relative incidences of SRBs when compared to lorazepam (HR 2.373 (95% CI [1.089, 5.165] *p* = 0.0021) and clonazepam (HR 2.670 (95% CI [1.430, 4.988] *p* = 0.0297).^18^ Midazolam was associated with reduced SRBs following FDR adjustment (*p*=0.0315).^18^

Independently, it was reported that in patients diagnosed with MDD, clonazepam was not associated with a reduction in suicidality as indicated by the HAM-D.^19^

### Non-benzodiazepine gamma-aminobutyric acid (GABA)ergic sedative-hypnotics

We identified four studies that reported on the association between non-benzodiazepine GABAergic sedative-hypnotics and measures of suicidality. Studied agents include zolpidem (*n* = 2) and eszopiclone (*n* = 2).

Findings suggest that zolpidem reduces SI in insomnia disorder patients. It was also reported that improvement in SI was moderated by improvement in overall insomnia.^11^^,^^20^ In patients exhibiting SI, insomnia, and depression, it was observed that zolpidem administration was associated with reduced long-term insomnia, which was, in turn, associated with a reduction in suicidal thoughts (Longitudinal linear association (beta) = 0.12, standard error (SE) = 0.04, *p* = 0.002).^11^ Likewise, in an exploratory analysis using the same population, it was observed that zolpidem was not associated with a reduction in the SSI; however, a reduction in scores was significantly positively correlated to the improvement in insomnia (Longitudinal effect [autoregressive covariance] = 9.78, *p* = 0.002).^20^ Zolpidem was associated with a greater reduction in the C-SSRS SI scores in participants with severe baseline insomnia (−0.41 ± 0.21) versus those with mild–moderate baseline insomnia (−0.08 ± 0.15),^11^ as measured by the Insomnia Severity Index (ISI).^11^

Independently, in patients exhibiting both MDD and insomnia, eszopiclone + fluoxetine combination therapy was not found to be associated with an increased risk of suicidality relative to placebo + fluoxetine.^21^^,^^22^

### Orexin receptor antagonists

We identified 8 studies reporting on the association between ORAs and suicidality. Reported medications include daridorexant, lemborexant, and suvorexant. All RCTs assessed safety outcomes associated with ORAs wherein suicide-related outcomes were included as a safety measure.

Taken together, there was no increase in SI or SA. In patients diagnosed with insomnia, dariorexant, lemborexant, and suvorexant were not associated with an increase in SI or SA.^23^^,^^24^^,^^25^ Likewise, in patients diagnosed with both Alzheimer’s disease and sleep–wake rhythm disorder, lemborexant was not associated with an increase in suicidality as measured by the C-SSRS.^26^

### Cognitive behavioral therapy for insomnia

Four studies investigated the anti-suicidal effects of CBT-I.

Studies suggest that CBT-I is effective in reducing SI. It was reported that CBT-I was associated with a reduction in SI in patients diagnosed with insomnia (Linear mixed model analysis (*t*) = −3.35, *p* = 0.001).^14^ Likewise, using the distribution of the PRODuct Confidence Limits for INdirect effects (PRODCLIN) program, it was observed that improvement in SI was moderated by an improvement in insomnia symptoms. (Estimate of indirect effect (*αβ*) = −3.20 (95% CI [−5.74, −0.87])).^12^ Group CBT-I is associated with reduced SI (Effect size (*d*) = −0.64, *p* = 0.01)^15^; however, unguided, internet-based CBT-I transiently demonstrated a reduction in suicidal thoughts post-intervention (*d* = 0.13, *p* = 0.007), and not after a 6-month follow-up (*d* = 0.08, *p* = 0.303).^27^

## Discussion

This systematic review provides the most recent assessment of sleep-related interventions on suicidality outcome measures. Overall, zolpidem and CBT-I are associated with a reduction in SI.^11^^,^^12^^,^^14^^,^^20^ Alprazolam and midazolam are associated with reduced risk of SI, SA, and SC in comparison to lorazepam and clonazepam.^17^ Similarly, ORAs are not associated with an increase or decrease in SI or SA.^23^^,^^24^^,^^25^^,^^26^ Our findings are in accordance with additional reviews suggesting that emerging evidence suggests that sleep interventions can be beneficial for suicidality; however, additional studies in more diverse populations, especially those highly comorbid with sleep disorders (e.g. substance use disorder, attention-deficit/hyperactivity disorder [ADHD]) are needed.^28^^,^^29^

Moreover, findings suggest that the efficacy of sedative-hypnotics and CBT-I in reducing SI are moderated by a reduction in insomnia symptoms. For example, zolpidem-mediated anti-suicidal effects were moderated as a function of changes in insomnia symptoms.^10^ Likewise, CBT-I displays SI reductive effects in insomnia patients, moderated by an improvement in insomnia symptomatology.^14^ However, the anti-suicidal effects of sedative-hypnotics are not entirely accounted for by improvements in insomnia, as alprazolam and midazolam broadly reduce SI, SA, and SC in patients diagnosed with PTSD.^17^ Likewise, zolpidem’s anti-suicidal effects are associated with an improvement in depression, suggesting an underlying pleiotropic mechanism.^10^

Our findings have both clinical and research implications. Firstly, insomnia is established as a risk factor for suicidality.^30^^,^^31^ Practitioners should be evaluating individuals presenting with psychiatric and medical disorders as to whether they are experiencing insomnia and should it be prioritized as a therapeutic target. Notwithstanding important safety information communicated by regulators and present package inserts alerting practitioners to the potential of suicidality associated with sedative-hypnotics, we did not identify a replicated body of literature documenting an increase in suicidality associated with any agent. Moreover, in many cases, either no effect or decreased ratings across aspects of SI and behavior were noted. In addition, CBT-I also, perhaps by improving symptoms of insomnia, manifests benefits across aspects of suicidality. From a research perspective, discerning neurobiological and cognitive mechanisms that link insomnia to aspects of suicidality is a priority vista. For example, it could be hypothesized that chronobiological disturbances are linked to changes in neurobiology that affect aspects of cognition and reward valence that may portend aspects of suicidality.^30^^,^^31^

Several limitations affecting our inferences and interpretations should be noted. First, inconsistent definitions of the aspects of suicidality, as well as disparate measures of these dimensions were applied between studies, potentially affecting the internal consistency of our findings. Second, the heterogeneity of duration and enrollment populations affect the predictive validity of our data. Studies also varied with respect to whether aspects of suicidality were a safety measure or an efficacy outcome. Third, there were insufficient data concerning participant history of mental disorders and/or suicide. Fourth, studies varied in dosing regimens as well as the non-pharmacological interventions participants were receiving while enrolled in respective studies.

Taken together, our systematic review summarizes the extant literature evaluating the anti-suicidal effects of sedative-hypnotics and/or CBT-I. In turn, highlighting the efficacy treating comorbid insomnia has in reducing suicidality. Available evidence suggests that FDA-approved sleep aids do not increase suicidal risk. Further research should aim to identify the most effective ways to optimize the anti-suicidal effects of sedative-hypnotics and/or CBT-I, potentially through clarifying the mechanisms the aforementioned interventions influence when reducing SI.

## Supporting information

Valentino et al. supplementary material Valentino et al. supplementary material

## Supplementary material

The supplementary material for this article can be found at http://doi.org/10.1017/S1092852925000318.
