# Supplementary material for: A systematic review of anti-suicidal effects of sedative-hypnotics and cognitive behavioral therapy for insomnia
Source: CNS Spectr. 2025 Jun 2;30(1):e45. doi: 10.1017/S1092852925000318 (PMC13064792; doi:10.1017/S1092852925000318)
Supplement: Valentino et al. supplementary material [file S1092852925000318sup001.docx]

### **eTable 1. Search terms**

| **Term Group** | **Search String** | **Number of results on July 30, 2024** |
| --- | --- | --- |
| Benzodiazepines | (Suicide or suicidal) and (Benzodiazepine or alprazolam or brotizolam or midazolam or triazolam or estazolam or loprazolam or lorazepam or lormetazepam or temazepam or flunitrazepam or flurazepam or nitrazepam or quazepam or olanzapine) | 3740 |
| Z-Drugs | (Suicide or suicidal) and (Z-drug or Z drug or Z-hypnotic or Z hypnotic or Zaleplon or Zolpidem or zopiclone or eszopiclone or Ambien or Ambien CR or Edluar or Zolpimist or Sonata or Lunesta) | 937 |
| Orexin Receptor Antagonists | (Suicide or suicidal) and (orexin antagonist or orexin receptor antagonist or daridorexant or suvorexant or lemborexant or seltorexant) | 207 |
| Other FDA Approved Sedative-Hypnotics | (Suicide or suicidal) and (Doxepin or secobarbital or benadryl or diphenhydramine or unisom or doxylamine) | 2710 |
| Cognitive Behavioral Therapy | (Suicide or suicidal) and (CBT or Cognitive Behavioral Therapy or Cognitive Behavioural Therapy) | 6016 |
